# Reference method matters: DXA system differences influence agreement with 3D optical imaging body composition estimates

**DOI:** 10.1038/s41366-026-02148-6

**Published:** 2026-07-07

**Authors:** Sophia Ramirez, Miriam Bustami, Christian Rodriguez, Sri Lakshmi S. Devarakonda, Shengping Yang, Diana M. Thomas, Grant M. Tinsley, Steven B. Heymsfield

**Affiliations:** 1 Pennington Biomedical Research Center, LSU System, Baton Rouge, LA USA; 2 Department of Mathematical Sciences, United States Military Academy, West Point, NY USA; 3 Department of Kinesiology & Sport Management, Texas Tech University, Lubbock, TX USA

**Keywords:** Medical research, Translational research

## Abstract

**Background:**

Dual-energy X-ray absorptiometry (DXA) is often used as the reference method for calibrating and validating other clinically useful body composition devices, although previous studies report DXA system differences in component estimates. This study evaluated the hypothesis that body composition estimates, specifically percent body fat (BF%), acquired with a method such as 3D optical (3DO) imaging might lead to varying conclusions when judged against different DXA systems and software versions.

**Methods:**

BF% was evaluated in healthy adult participants with a 3DO imaging system (Size Stream, Mobile Fit) and three DXA systems (Hologic Horizon A [Apex 4.0.2] and Discovery A [Apex 5.6.1.3]), and GE Lunar iDXA [enCore 13.60.033] on the same day. Agreement between BF% estimates by 3DO and each DXA system was evaluated with multiple statistical procedures.

**Results:**

Ninety-nine participants completed all four evaluations. Linear correlations (r and R^2^s), Bland–Altman analyses, mean differences, mean absolute errors, root-mean square errors, and concordance correlations differed between %BF by 3DO and each of the DXA devices. Relative to BF% by 3DO, agreement measures were best for Discovery A compared to the other two DXA scanners. A linear mixed-effects model with method as a fixed effect and participant as a random intercept revealed a statistically significant effect of assessment method on estimated BF%: Horizon A DXA (X ± SD, 36.4 ± 9.3%) differed significantly from 3DO (33.3 ± 8.8%), iDXA (35.2 ± 10.9%), and Discovery A (34.4 ± 10.0%); and iDXA and Discovery A both differed significantly from 3DO and Horizon A DXA. Significant interactions between method and sex and method and BMI category were also observed, indicating the differences between measurement methods were not uniform across males and females or BMI categories.

**Conclusions:**

Our findings indicate that conclusions related to a clinical body composition method’s accuracy depend on the specific DXA device and associated software version chosen to serve as the reference method.

## Introduction

Increasing attention is focused on practical, low cost, and accessible methods for evaluating adiposity in clinical settings [1]. These methods primarily include conventional anthropometry, bioimpedance analysis (BIA), three-dimensional optical (3DO) imaging, and ultrasound. All of these methods all share evaluation of physical properties (e.g., circumferences, resistance, reactance, tissue depth) that are used with prediction equations to estimate the mass of selected components such as fat, lean soft tissue, and bone mineral content [2]. These methods and their predictions are usually validated by investigators when applied in new settings, samples, or patient groups. A widely used validation approach is to compare method-acquired component estimates against ground-truth measurements; the latter, for practical reasons, is usually dual-energy X-ray absorptiometry (DXA) [2–4]. A foundational assumption of this approach is that DXA systems provide accurate criterion estimates independent of device manufacturer, software version, or system settings. However, several studies over the past decade have reported between-DXA system differences in body composition estimates [5–7]. These discrepancies raise an important concern: conclusions regarding the validity of emerging body composition methods may depend on the specific DXA system applied as the reference. We therefore hypothesized that body composition estimates acquired with a method such as 3DO imaging might lead to varying conclusions when judged against different DXA systems and software. The aim of the present study was to assess this hypothesis by evaluating the validity of 3DO imaging for estimating percent body fat (BF%) across three different DXA instruments.

## Methods

### Participants

The study aim was conducted in a cross-sectional sample of healthy adults evaluated between January 2023 and January 2024 at the Body Composition Laboratory of Pennington Biomedical Research Center (PBRC), Baton Rouge, Louisiana, USA. This report extends an earlier unrelated publication that includes data presented in the analyses that follow [8]. The aim of that publication was to report cross-calibration equations across DXA scanners. Participants were healthy adults (*N* = 101) varying in age and body mass index (BMI). Eligibility criteria included being in good health, non-pregnant, and below the weight limit of the DXA scanners (<200 kg). All participants provided written informed consent prior to participation. The study protocol was approved by the institutional review board of PBRC (IRB #1016-0000), and all procedures were conducted in accordance with the Declaration of Helsinki.

### Study visit procedures

Participants arrived at the research facility following an overnight fast of ~10 h. Participants were allowed to take prescription medications prior to the visit and were asked to refrain from ingesting any calcium supplements 30 min prior to evaluation [9] or having procedures involving barium contrast agent ingestion. Height and body weight were measured using a calibrated stadiometer and scale prior to body composition assessment. Female participants of childbearing potential completed a urine pregnancy test within 72 h of DXA scanning, with a required negative result prior to imaging.

### Dual-energy X-ray absorptiometry

Whole-body DXA scans were performed to assess fat, lean soft tissue, and bone mineral content. Percent of body weight as fat (%BF) was derived using the measured DXA body weight. Scans were conducted using Hologic Horizon A (Apex version 4.0.2) and Discovery A (Apex 5.6.1.3) scanners (Hologic Inc., Bedford, MA) and a GE Lunar iDXA (enCore 13.60.033) scanner (GE Healthcare, Milwaukee, WI). Both Hologic scanners were set on the Classic Calibration mode.

All DXA scans were performed by a trained and certified technician following manufacturer-recommended positioning and scanning protocols. Participants were scanned in light clothing, free of metal objects, and positioned supine with standardized limb placement. Daily quality control and calibration procedures were performed using manufacturer-supplied phantoms to ensure scanner stability and accuracy throughout the study period.

Whole-body scans were analyzed using manufacturer proprietary software to derive body composition outcomes.

### Three-dimensional optical imaging

Three-dimensional optical imaging assessment was conducted via a mobile phone application that produces a 3D avatar from two individual 2D images (frontal and lateral) from a mobile device camera (Mobile Fit, Size Stream, Cary, NC, USA). The comparable optical imaging algorithm (MeThreeSixty, Size Stream, Cary, NC, USA) showed excellent agreement with waist, hip, thigh, and arm circumferences relative to expert flexible tape-measured circumferences [10]. Participants were positioned for the frontal image holding an A-pose (i.e., feet spread shoulder width apart, and arms straightened and raised laterally), while the lateral image consisted of participants keeping their arms at their sides with their feet close together. The participants’ right sides were evaluated for the lateral images. Scans were performed using an iPad (9th generation) mounted on a tablet stand. The stand was positioned on the floor to allow for participant positioning as indicated by an on-screen body outline.

From the reconstructed 3D body model, automated proprietary algorithms were used to extract anthropometric measures, including circumferences of the waist, hip, arms, and legs along with other body shape features relevant to body composition estimation. Percent of body weight as fat was derived by scanner software using proprietary algorithms. The resulting BF% from these proprietary algorithms were then compared to BF% estimated by the three aforementioned DXA technologies. All scans were visually screened for completeness and quality prior to analysis. If motion artifacts or positioning errors were identified, scans were repeated when feasible.

### Statistical methods

The first series of analyses evaluated the relationships between BF% derived from 3DO and each DXA system using simple linear regression analysis, including Pearson’s correlation coefficients (r values). Corresponding Bland–Altman plots were generated, in conjunction with mean differences (MD), 95% limits of agreement (LOA), and linear regression, to allow for the evaluation of proportional bias and individual-level accuracy [11]. Measures of agreement between 3DO optical BF% estimates and DXA systems were derived including Pearson’s R^2^. root mean square error (RMSE), mean absolute error (MAE), and Lin’s concordance correlation coefficient (CCC). The second series of analyses evaluated if significant differences in BF% estimates were present across the four methods, 3DO and the three DXA systems, and if sex, age, and BMI impacted these differences. A linear mixed-effects model was used to assess the effects of measurement method, sex, age, and BMI category on BF% estimates, with repeated measures accounted for by including a random intercept for each participant. BMI was categorized into four groups using standard cutoffs: Normal (<25.0 kg/m²), Overweight (25.0–29.9 kg/m²), Obesity I (30.0–34.9 kg/m²), and Obesity II+ (≥35.0 kg/m²). Age and BMI were mean-centered prior to analysis to improve interpretability of model coefficients. Model residuals were examined visually using residual plots and quantile–quantile plots to assess assumptions of homoscedasticity and normality.

Estimated marginal means were calculated for the main effect of method as well as method-by-sex and method-by-BMI category interactions using the emmeans R package. Pairwise comparisons between methods were adjusted for multiple comparisons using Tukey or Sidak corrections as appropriate. Compact Letter Displays (CLDs) were generated to summarize statistically significant differences among methods across participant subgroups. All analyses were conducted in R (version 4.5.1) using the nlme, emmeans, and dplyr packages. Statistical significance was accepted at α = 0.05.

## Results

**Fig. 1:**
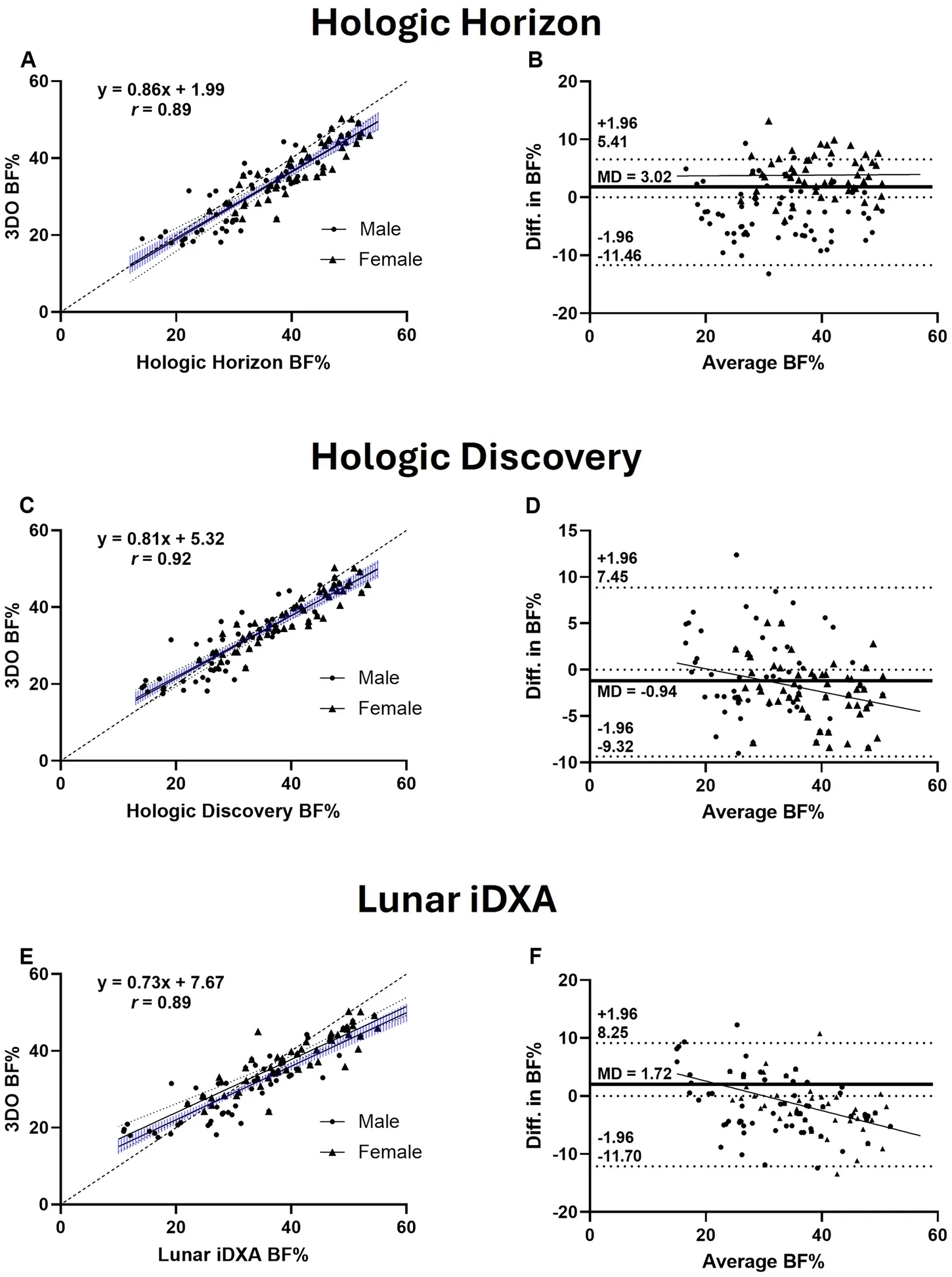
3D optical (3DO)–predicted body fat percentage (BF%) versus DXA-derived BF% across the three DXA platforms on the left and corresponding Bland–Altman on the right with difference in BF% (DXA-3DO) plotted against average BF%. Linear regression models and r values are shown in each of the scatter plots on the left. All three regression analyses were statistically significant at *p* < 0.001; dashed lines are identity. Slopes of Bland–Altman plots are shown in Table 1 dashed lines are mean ±1.96 SD and bold horizontal lines are the mean differences (MD) between DXA-measured BF% and 3DO-BF%. **A**, **B** Hologic Horizon; **C**, **D**, Hologic Discovery; and **E**, **F**, Lunar iDXA scanners.

**Fig. 2:**
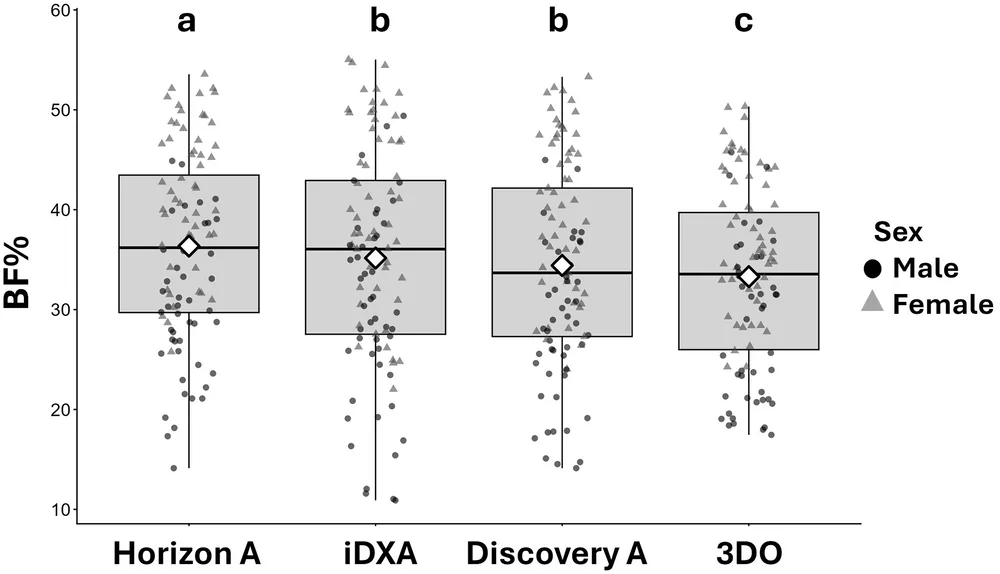
Inter- method comparison of body fat % estimates. Methods are ordered from highest to lowest by their respective mean estimates. Methods sharing the same letter (a, b, or c) were not significantly different from each other. Methods not sharing the same letter (a, b, or c) were significantly different from each other. Circles indicate individual participant data, and white diamonds indicate mean values.

A total of 101 participants were enrolled in the study; of these, the final analysis included 99 participants (X ± SD, age 49.7 ± 16.0 y [range, 20–78 years], BMI 29.5 ± 7.0 kg/m^2^ [18–49 kg/m^2^], and sex 53.5% female) that completed all four scans. Scatter plots and corresponding Bland–Altman analyses are shown in Fig. 1. Linear regression analyses demonstrated a strong relationship between 3DO–predicted BF% and DXA-derived BF% for all three DXA systems (all *p* < 0.001). The strongest association was observed for the Discovery A system (*r* = 0.92), while iDXA and Horizon A systems showed similar correlations (*r* = 0.89 for both). Bland–Altman analyses revealed significant bias for Discovery and iDXA systems (slopes for BF% difference (3DO-DXA) vs. average BF%, −0.12 and −0.21, *p* < 0.05) while the comparable slope for the Horizon system (−0.04) was non-significant. Mean differences between 3DO and DXA BF% ranged from −1.1%, 1.82%, and −3.03% for Discovery A, iDXA, and Horizon A scanners, respectively (Table 1). Corresponding LOAs were ±7.86%, ±8.15%, and ±9.65% for Discovery A, Horizon A, and iDXA scanners, respectively. Good agreement between 3DO–predicted BF% and DXA-derived BF% was exhibited across all three DXA platforms (Table 1). Mean absolute errors ranged from 4.2% for Discovery A to 5.5% for Horizon A; and RMSE ranged from 7.6% for Discovery A to 8.8% for Horizon A. Concordance correlation coefficients indicated strong agreement, with values of 0.84 for Horizon A, 0.91 for iDXA, and 0.93 for Discovery A (Table 1). Pearson’s R^2^ was highest for Discovery A (0.84) and the same for Horizon A and iDXA (0.80). Of the agreement measures for 3DO BF% (r’s, MDs, R^2^s, MAEs, RMSEs, and CCs), Discovery A ranked first in all of them, iDXA ranked second except for a tie with Horizon A for r and R^2^, and Horizon A ranked last. Three-dimensional optical predicted BF% values were next compared to BF% estimates for the three DXA systems. Across the 99 participants, a linear mixed-effects model with method as a fixed effect and participant as a random intercept revealed a statistically significant effect of assessment method on estimated BF% (F (3, 281) = 25.6, *p* < 0.0001). Diagnostic checks confirmed that model assumptions of normality, homoscedasticity, and independence of residuals were not violated. Follow up testing using Tukey-adjusted pairwise comparisons demonstrated significant differences in BF% between individual methods (Fig. 2): Horizon A DXA (X ± SD, 36.4 ± 9.3%) differed significantly from 3DO (33.3 ± 8.8%), iDXA (35.2 ± 10.9%), and Discovery A (34.4 ± 10.0%); and iDXA and Discovery A both differed significantly from 3DO and Horizon A DXA.

**Table 1:** Measures of agreement between 3DO BF% estimates and DXA systems.

DXA system	R^2^	MD (%)	MAE (SD, %)	RMSE (%)	CCC	LOA (%)	BA Slope
Hologic Horizon	0.80	−3.03	5.5 ± 6.8	8.8	0.84	±8.15	−0.04
Hologic Discovery	0.84	−1.1	4.2 ± 6.3	7.6	0.93	±7.86	−0.12^a^
GE Lunar iDXA	0.80	−1.82	5.1 ± 6.8	8.5	0.91	±9.65	−0.21^a^

*BA* Bland–Altman, *BF*% percent body fat, *CCC* concordance correlation coefficient, *CE* constant error, *LOA* limits of agreement, *MAE* mean absolute error, *RMSE* root-mean square error.

^a^Slope significant at *p* < 0.05.

Significant main effects were also observed for Sex (F(1, 95) = 173.52, *p* < 0.0001), age (F (1, 95) = 60.89, *p* < 0.0001), and BMI category (F (3, 95) = 66.26, *p* < 0.0001), reflecting expected differences in %BF by participant characteristics. Notably, significant interactions existed between method and sex (F (3, 281) = 4.34, *p* = 0.0052) and between method and BMI category (F (9, 281) = 4.09, *p* < 0.0001), indicating that the differences between measurement methods were not uniform across males and females or BMI categories. The interaction between method and age approached significance (F (3, 281) = 2.59, *p* = 0.053), suggesting a possible, though weaker, age-dependent effect on method differences.

## Discussion

The current study was founded on the observation that DXA systems vary in their estimates of major body components such as fat, lean soft tissue, and bone mineral mass [5, 7, 8]. From those earlier findings, we hypothesized that referencing clinically applicable methods such as 3DO imaging against DXA as the “gold standard” may not lead to one conclusion regarding agreement for measures such as BF%. Specifically, we observed in the current study that BF% estimated by a 3DO application varied in level of agreement across three widely used DXA systems. Commonly used measures of agreement (r’s, MDs, R^2^s, MAEs, RMSEs, CCs) consistently showed improved performance for the Discovery A system as compared to the iDXA and Horizon A systems. Had we used only one of these three systems in a validation study we might have come to different conclusions regarding the accuracy of the 3DO application for estimating BF%, depending on which system was chosen.

Many studies have reported agreement between clinically applicable body composition methods such as 3DO, BIA, ultrasound, and anthropometry and DXA. The observed agreement for the specific device or instrument can influence how the method is perceived and adopted in scientific and medical communities. While the observed differences in DXA system agreement in the current study were generally small, such observations can promote inaccurate ranking of method accuracy, particularly when incorporated into systematic reviews and meta-analyses.

Several important implications arise from our observations. First, investigators evaluating clinically applicable methods, as in the current study should provide details in their published report related to the applied DXA system such as software version and any other specific settings or acquisition procedures. These details are often overlooked in related publications, thus making it difficult to replicate observations. Second, the use of proprietary algorithms in both DXA and 3DO systems limits direct insight into the sources of observed discrepancies and ideally manufacturers should disclose their calibration methods. An alternative and more scalable approach is the development of standardized cross calibration datasets. Rather than requiring access to proprietary algorithms, investigators could evaluate device outputs on a shared reference population measured across multiple platforms, enabling empirical mapping and harmonization between systems [8, 12]. Such an approach would allow for the construction of conversion models and bias corrections that account for systematic and proportional differences without reliance on manufacturer transparency. Establishing and maintaining reference datasets will require a coordinated effort from the research community but would provide a practical framework for ensuring comparability across studies, improving meta-analytic validity, and advancing the field toward device-agnostic body composition assessment [13]. Third, certain measurement techniques may systematically over- or under-estimate %BF depending on sex and BMI, underscoring the importance of considering participant characteristics when comparing body composition methods. Therefore, it is important to assess the validity of selected body composition methods against criterion methods and models. While some methodological questions remain unresolved, a relative consensus exists that multi-component models, notably four-compartment models, are accepted in vivo reference methods for estimation of body composition at the molecular level. Using specific input variables, most commonly body mass, volume, water, and bone mineral content, whole-body FM and FFM can be accurately estimated. Lastly, the concepts advanced in the current study apply to other commonly used measures in obesity research. For example, resting energy expenditure (REE) prediction models often include DXA-measured fat and fat-free mass as covariates [14]. Variability in REE predictions across different models might thus partly reflect the specific DXA system used to assess body composition.

In sum, we availed ourselves in the current study of three available DXA systems to assess the hypothesis that body composition estimates acquired with a method such as 3DO imaging might lead to varying conclusions when judged against different DXA systems and software. Our findings bring attention to the observation that not all DXA systems provide identical body composition results, and these differences can translate into how the validity of clinically applicable methods are perceived. Moreover, these findings emphasize the importance of providing DXA system details in publications related to body composition validation studies and for manufacturers to be transparent in reporting how their system prediction equations were developed.

## Data Availability

Data described in the manuscript will be made available upon reasonable request pending application and approval by the investigators.
